# Increased number of circulating exosomes and their microRNA cargos are potential novel biomarkers in alcoholic hepatitis

**DOI:** 10.1186/s12967-015-0623-9

**Published:** 2015-08-12

**Authors:** Fatemeh Momen-Heravi, Banishree Saha, Karen Kodys, Donna Catalano, Abhishek Satishchandran, Gyongyi Szabo

**Affiliations:** Department of Medicine, University of Massachusetts Medical School, LRB208, 364 Plantation Street, Worcester, MA 01605 USA

**Keywords:** Acute alcoholic hepatitis, Alcohol, Liver injury, Extracellular vesicles, Exosomes, Microvesicles, Biomarker, Liquid biopsy, Human, miRNA

## Abstract

**Background:**

It has been well documented that alcohol and its metabolites induce injury and inflammation in the liver. However, there is no potential biomarker to monitor the extent of liver injury in alcoholic hepatitis patients. MicroRNAs (miRNAs) are a class of non-coding RNAs that are involved in various physiologic and pathologic processes. In the circulation, a great proportion of miRNAs is associated with extracellular vesicles (EVs)/exosomes. Here, we hypothesized that the exosome-associated miRNAs can be used as potential biomarkers in alcoholic hepatitis (AH).

**Methods:**

Exosomes were isolated from sera of alcohol-fed mice or pair-fed mice, and plasma of alcoholic hepatitis patients or healthy controls by ExoQuick. The exosomes were characterized by transmission electron microscopy and Western blot and enumerated with a Nanoparticle Tracking Analysis system. Firefly™ microRNA Assay was performed on miRNA extracted from mice sera. TaqMan microRNA assay was used to identify differentially expressed miRNAs in plasma of cohort of patients with AH versus controls followed by construction of receiver operating characteristic (ROC) curves to determine the sensitivity and specificity of the candidates.

**Results:**

The total number of circulating EVs was significantly increased in mice after alcohol feeding. Those EVs mainly consisted of exosomes, the smaller size vesicle subpopulation of EVs. By performing microarray screening on exosomes, we found nine inflammatory miRNAs which were deregulated in sera of chronic alcohol-fed mice compared to controls including upregulated miRNAs: miRNA-192, miRNA-122, miRNA-30a, miRNA-744, miRNA-1246, miRNA 30b and miRNA-130a. The ROC analyses indicated excellent diagnostic value of miRNA-192, miRNA-122, and miRNA-30a to identify alcohol-induced liver injury. We further validated findings from our animal model in human samples. Consistent with the animal model, total number of EVs, mostly exosomes, was significantly increased in human subjects with AH. Both miRNA-192 and miRNA-30a were significantly increased in the circulation of subjects with AH. miRNA-192 showed promising value for the diagnosis of AH.

**Conclusion:**

Elevated level of EVs/exosomes and exosome-associated miRNA signature could serve as potential diagnostic markers for AH. In addition to the biomarker diagnostic capabilities, these findings may facilitate development of novel strategies for diagnostics, monitoring, and therapeutics of AH.

**Electronic supplementary material:**

The online version of this article (doi:10.1186/s12967-015-0623-9) contains supplementary material, which is available to authorized users.

## Background

Alcoholic hepatitis (AH) is an acute hepatic inflammation accompanied with substantial morbidity and mortality and increasing health problems across the world. AH is a result of the complex interactions between ethanol metabolism, inflammation, and innate immunity on the liver [1]. Currently the enzymatic activity of alanine aminotransferase (ALT) and aspartate aminotransferase (AST) in blood are the most commonly used indicators for detection of liver damage; however, increased serum ALT- and AST are not specific for the type of liver disease, the extent of liver damage or the absence or presence of liver inflammation. In addition, ALT/AST increase is not specific to AH and occurs in many other liver disorders [2, 3]. Moreover, these two diagnostic markers do not always correlate well with the disease stage and extent of hepatocellular damage or liver fibrosis [3].

MicroRNAs (miRNAs) are small (17–25 nucleotide long) non-coding RNAs that regulate gene expression post-transcriptionally [4, 5]. miRNAs play important roles in different pathogenesis pathways and can be deregulated in different diseases. A growing body of evidence shows that miRNAs can act as novel biomarkers for diagnosis and therapeutic monitoring of various diseases such as cancers, neurodegenerative disorders, heart disease, and infection [5–8]. miRNAs play a substantial role in the regulatory gene expression process and it is estimated that about one-third of the human genome transcriptome being regulated by actions of miRNAs [9]. miRNAs influence gene regulation of essential biological pathways such as cellular development, proliferation, apoptosis, and cellular signaling [5, 10]. From its intracellular origin, miRNA can be secreted extracellularly bound to AGO2 [10], or secreted in cell-derived extracellular vesicles/exosomes and serve as personalized signatures reflecting the disease status or perform cell-to-cell communication [11].

Differential expression signatures of miRNAs associated with different diseases have been evaluated mostly in whole plasma and serum [12, 13]. However, it can be difficult to interpret the result of disease-specific miRNA biomarkers in healthy and diseased patients due to genetic material instability stemming from RNases, sample variation, normalization, and nonspecific activated pathways in various diseases [14]. With the introduction of EVs as an enriched source of miRNAs, new horizons opened in biomarker discovery based on EVs [15]. EVs are a mixed population of heterogeneous membranous vesicles shedding from cells in both physiological and pathological states. Their sizes range from 50 nm in diameter and up to 1,000 nm [10]. Exosomes are the smallest subpopulation of EVs originating from multivesicular bodies and can be isolated from different biofluids using standardized protocols. Exosomes can act as stable reservoirs of biomarkers in different diseases by containing genetic materials that reflect the disease stage [16].

A great proportion of circulating miRNA is associated with exosomes. Exosome associated miRNA is reported to be more stable and resistant to RNase enzymatic activity compared to non-exosome associated miRNAs [17, 18]. Although there is some evidence indicating deregulation of miRNAs in different liver diseases [19, 20], there is no study regarding the biomarker value of exosome-associated miRNAs in AH. The aim of the present study was to assess the utility of exosome-associated miRNAs as potential biomarkers for AH. In the present study, we demonstrate that miRNAs in circulation are largely encapsulated in exosomes and carry a specific signature after alcohol induced liver injury in chronic alcohol fed mice and patients with AH. We used chronic alcohol feeding in a mouse model and found a specific signature of miRNA associated with liver injury. Next, we validated our results in a cohort of human subjects with AH. Our results indicate that exosomes transport alcohol induced liver injury-specific molecular information in the circulation.

## Methods

### Human studies

Confirmed cases of alcoholic hepatitis (n = 14) and healthy individuals (n = 10) were enrolled in the study. The diagnosis of alcoholic hepatitis was established by expert clinicians based on medical history, physical examination and laboratory data according to the guidelines of the American College of Gastroenterology [21]. In cases of uncertain diagnosis, liver biopsy was obtained. Patients were included from different stages of alcoholic hepatitis. Healthy individuals were defined as being free of any systemic and non-systemic diseases based on patients’ history and routine laboratory findings identified by primary care physician. These individuals provided history of “only social” alcohol consumption. Both controls and patients were enrolled consecutively to study to avoid selection bias. The study protocol was approved by Institutional Review Board for the Protection of Human Subjects in research at the University of Massachusetts Medical School (Worcester, MA, USA). Written informed consents were obtained from all subjects.

### Animal studies

6 to 8-week-old female C57BL/6 mice were purchased from Jackson Laboratory. The animal study protocol was approved and conducted according to the regulations of the Institutional Animal Care and Use Committee (IACUC) of the University of Massachusetts Medical School (Worcester, MA, USA). In the alcohol experimental group (n = 12), mice received 5% (v/v) ethanol (36% ethanol-derived calories) containing Lieber–DeCarli diet (EtOH) for 4 weeks. The matched pair-fed animals received (n = 12) an isocaloric alcohol-free diet (pair-fed) to ensure equal nutrient and calorie intake between groups. Mice were euthanized at the end of experimental time course and serum was separated from the whole blood and the livers were collected. Serum ALT was measured using a kinetic method (D-Tek LLC).

For histopathological analysis, sections of formalin-fixed livers were stained with hematoxylin–eosin to study for steatosis, necrosis and inflammation. Flowcytometry was performed to study the immune cell population in the liver of the pair fed and ethanol fed mice as previously described [22].

### Exosomes isolation

For exosome isolation from sera of mice and plasma of human subjects, exosomes were isolated from 150 µl of sera or plasma following the previously established protocol [17]. Briefly, the serum or plasma were centrifuged at 1,500×*g* for 5 min to remove cells and 10,000×*g* for 20 min to remove residual cellular debris. Samples were serially filtered through 0.8, 0.44 μm (Millipore, Billerica, MA, USA). The filtered supernatant was used to precipitate exosomes with Exoquick-TC™ (according to the manufacturer’s guidelines). Exosome pellet was re-suspended in PBS. For isolating smaller subpopulation of exosomes (diameter <200 nm) we added an additional step before re-suspension in PBS and filtered the samples with 0.2 µm filters.

### Nanoparticle Tracking Analysis (NTA)

The concentration and size range of circulating EVs in human subjects and mice were identified by a NanoSight NS300 system (NanoSight, Amesbury, UK) supplied with a fast video capture and Nanoparticle Tracking Analysis (NTA) software. Before performing the experiments, the instrument was calibrated with 100 nm polystyrene beads (Thermo Scientific, Fremont, CA, USA). The samples were captured for 60 s at room temperature. NTA software processed the video captures and measured the concentration of the particles (particles/ml) and size distribution (in nanometer). Each specimen was measured three times.

### Electron microscopy

Isolated EVs from plasma and serum were re-suspended in PBS and placed on a formvar-coated copper grid and then allowed to settle for 45 min. Sequential PBS washing of the grid was done by positioning droplets of PBS on the top and applying absorbing paper in between. The samples were fixed by positioning the grid on the top of 2% paraformaldehyde placed on the parafilm for 15 min. After fixation and three washing steps, samples were contrasted by adding drops of 2% uranyl acetate for 15 min. A drop of 0.13% methyl cellulose and 0.4% uranyl acetate was placed on the parafilm grid was incubated at the top for 10 min. The grid was visualized by a Philips CM10 transmission electron microscope and images were recorded using a Gatan CCD digital camera.

### Western blotting

After isolating exosomes from mice sera, presence of CD63 and GRP78 were determined with western blot, using our laboratory’s established protocol. Briefly, exosomes were lysed in RIPA buffer and run on a 15% polyacrylamide gel. Proteins were transferred to nitrocellulose membrane overnight then blocked for 3 h in blocking buffer (1× TBST with 5% w/v nonfat dry milk). Primary antibody against CD63 and GRP78 (Abcam, Cambridge, MA, USA) was used overnight at 4°C at 1:2,000 dilution in the blocking buffer. For detection, secondary goat anti-mouse HRP-linked antibody (Santa Cruz Biotechnology, Dallas, TX, USA) was used for 1 h at a dilution rate of 1:5,000 in blocking. The immunoreactive bands were visualized by a Clarity™ Western ECL substrate kit (BioRad) according to the manufacturer’s protocol and LAS-4000IR Ver.2.02 (Fujifilm, Valhalla, NY, USA). Mouse primary hepatocytes were used as negative control.

### RNA isolation

Isolated EVs from 150 μl of mice sera and patients’ plasma were lysed in 500 and 1,000 µl of QIAzol Lysis reagent, respectively. Total RNA was extracted using Direct-zol*™* RNA MiniPrep isolation kit (Zymo Research Corp, Irvine, CA, USA). The standard manufacturer’s protocol was followed, and extracted RNA was eluted with 25 μl of RNase-free water. The RNA was quantified using and NanoDrop 1000 and the quality of RNA was assessed by 260/280 and 260/230 ratios.

### Microarray analysis

In addition, the Firefly miRNA multiplex assay (Firefly™ bioworks, Cambridge, MA, USA) was used to profile EV-associated miRNA isolated from mice sera for a targeted list of 68 miRNAs. Total RNA (12 μl) was assayed by the Firefly standard protocol and analyzed with a Millipore Guava 8HT flow cytometer.

Data processing, analysis, and visualizations were performed using Firefly™ Analysis Workbench. The software was used to compare data quality with historical values, perform image processing, normalization, and transformation. Raw probe intensities were analyzed using Wilcoxon’s signed rank test. Signal intensities were transformed to log ratio for each probe on the alcohol group array to the counter parting probe pair on the control array. miRNA-15b was used for normalizing array data. Signal log ratio was computed by using a *t* test. After generating a list of differentially expressed miRNAs, down-stream analysis was performed. The identified miRNA were clustered in by K mean clustering and heat-map generated. The targets of miRNA-192 and miRNA-30a target were visualized using Cytoscape V2.7. miRDB, miRanda, and mirSVR databases were used for miRNAs target predictions.

### miRNA analysis

We used TaqMan^®^ miRNA Assays (Applied Biosystems, Catalog number: 4366597) for confirmation of microarray and evaluating the expression level of miRNA-122 (Applied Biosystems, Catalog number: A25576), miRNA-30a (Applied Biosystems, Catalog number: 4427975) and miRNA-192 (Applied Biosystems, Catalog number: A25576). Reverse transcription was performed using a TaqMan stem loop primer, TaqMan primers, 10 ng RNA, and miRNA reverse transcription kit in an Eppendorf Realplex Mastercycler (Eppendorf, Westbury, NY). The cycle was as follows: 30 min, 16°C; 30 min, 42°C; 5 min 85°C. The cDNA was mixed with TaqMan^*®*^ Universal PCR Master and quantitative real-time PCR was performed using a Bio-Rad CFX96 iCycler device. Synthetic *C. elegans*-miRNA-39 was spiked as external control during the total RNA isolation from human plasma samples. Consistent with the array findings, miRNA-15b was used for normalization of mice sera in confirmatory qPCR analysis. The amplification efficiency of qPCR was verified in our lab and it was very close to 100%. Samples were processed in duplicate and the relative expression level of specific miRNA calculated by 2^−ΔΔCt^ method. NormFinder was used to identify the suitable endogenous normalizer [23].

### Statistical analysis

Data are expressed as mean ± standard error (SE). For pair-wise comparisons, non-parametric Mann–Whitney test or parametric *t*-test were used based on underlying distribution. The ability to discriminate between the alcohol fed mice/alcoholic hepatitis patients and control groups was evaluated by the receiver operating characteristic curve, and the area under the curve (AUC) was calculated as a measure of test accuracy. For group-wise comparison, Kruskal–Wallis non parametric test or ANOVA parametric test were used and a *p* ≤ 0.05 was considered as statistically significant. Correlation of ALT with exosome numbers was identified by Spearman correlation test. For statistical analysis and heat map data visualization, GraphPad Prism v.4.03 (GraphPad Software Inc.) and Firefly™ Analysis Workbench software were used, respectively.

## Results

### Total number of EVs is increased in alcohol-fed mice compared to the pair-fed mice

**Fig. 1:**
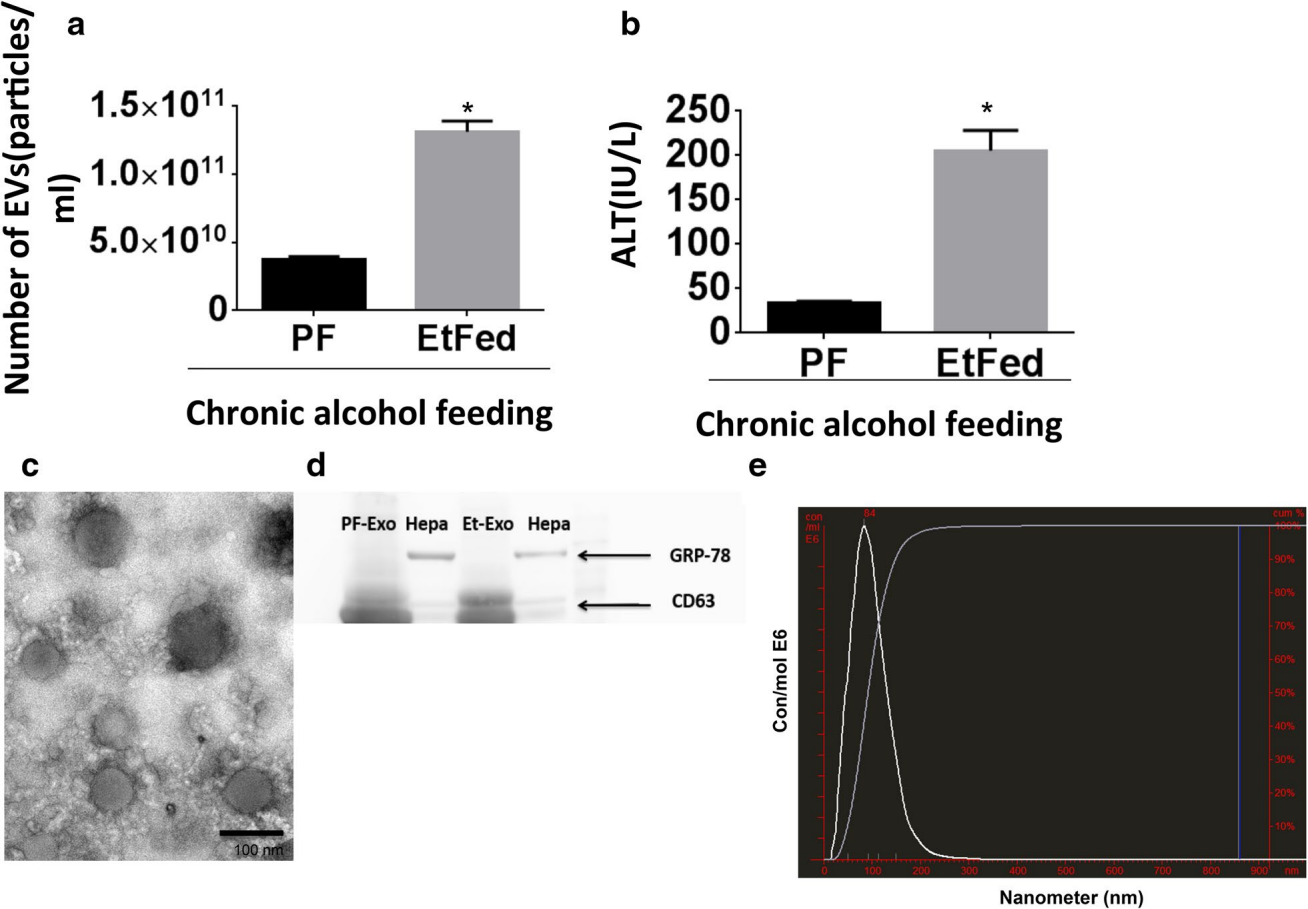
Extracellular vesicles (EVs)/exosomes characterization and enumeration and serum ALT levels. **a** Total number of EVs in the sera of chronic alcohol-fed mice and pair-fed mice were measured by a Nanoparticle Tracking analysis (Nanosight). **b** Serum ALT (IU/L) levels were measured in pair-fed mice and alcohol-fed mice. **c** Transmission electron micrographs of exosomes isolated from alcohol-fed mice showed diameters less than 150 nm. **d** Same amount of protein loaded into the all columns. Isolated exosomes from sera of alcohol-fed mice (Et-exo) and pair-fed mice (PF-exo) expressed CD63. GRP78 was detectable only in primary hepatocytes (Hepa). **e** The average size of exosomes was measured by Nanoparticle Tracking analysis (NTA).

The total number of EVs was significantly increased in mice sera after chronic alcohol feeding compared to pair-fed controls (p < 0.05), measured by NanoSight (Fig. 1a). Most of the EVs had a smaller diameter and were in the size of exosomes subfraction of EVs compared to the larger size microvesicles sub-fraction. Serum ALT levels were significantly increased after 4 weeks of alcohol feeding indicting alcohol-induced liver damage, as seen before in chronically alcohol-fed mice compared to the paired-fed controls (Fig. 1b) [24]. EVs isolated from alcohol-fed mice demonstrated the previously described morphology of EVs on electron micrograph [25], and expressed the exosomal marker CD63 (Fig. 1c, d). Loading of the same amounts of input proteins, exosomes isolated from sera of alcohol-fed mice and pair-fed mice showed similar amount of CD63 while primary hepatocytes showed less expression of CD63. GRP78, the intracellular endoplasmic reticulum marker, was present in primary hepatocytes while it was absent in the exosomes (Fig. 1d). Size distribution of the EVs in mice sera in both alcohol-fed mice and pair-fed mice indicated an abundance of smaller vesicles in the range of 50–150 nm supporting predominance of exosomes (Fig. 1e). Exosomes were isolated based on combination of serial filtration and precipitation reagent (ExoQuick). Quantitative measurement of particles with Nanoparticle Tracking Analysis (NTA) showed no significant difference in the distribution of EVs in crude serum and exosomes after isolation (data not shown). We used a 4-week Lieber–Decarli diet model for alcohol induced liver injury in mice which is an established model resulting in mild alcoholic steatohepatitis [26]. The histological and molecular changes confirmed liver injury, steatosis and inflammation in the Lieber Decarli model compared to pair-fed mice are shown in Additional file 1: Figure S1. Salient features included steatosis, macrophage/KC infiltration and activation, neutrophils activation and increased pro-inflammatory cytokines in the livers of alcohol-fed mice (Additional file 1: Figure S1).

### Correlation of ALT with exosome number

**Fig. 2:**
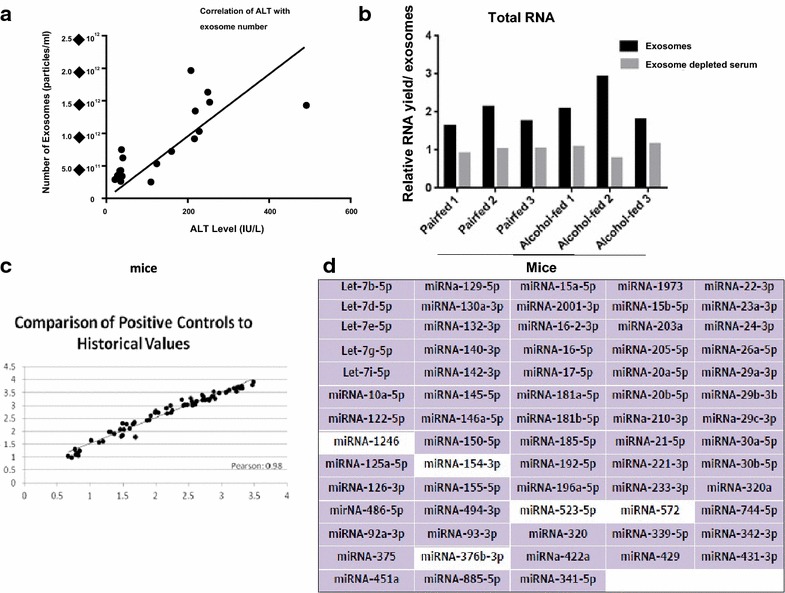
Correlation of exosome number with ALT, exosomes RNA yield and miRNA assay. **a** The correlation of exosome number (particle/ml) with ALT level (IU/L) was identified by Spearman's correlation test. **b** Exosomes were isolated and treated with RNase. The total amount of RNA in the exosome fraction and exosome-depleted serum was compared using a Nanodrop. **c** Historical values of spiked positive control miRNAs were compared with the assay values. **d** This graph depicts the miRNAs included in the whole inflammatory panel. *Purple color* indicates detectable miRNAs in our samples.

Exosome number (particles/ml) showed a strong and statistically significant correlation with ALT levels (IU/L) (Spearman’s Rho, r = 0.80) (p < 0.05) (Fig. 2a). Interestingly, a great portion of total serum RNA were packed within the serum-exosomes compared to the exosome depleted serum (Fig. 2b).

### Introducing endogenous control normalizer in EVs isolated from mice sera

Next, liver miRNA microarray was performed on exosome-associated miRNA, using The Firefly™ microRNA Assay which includes miRNA signatures of 68 miRNAs associated with liver inflammatory pathways. First to ensure reproducibility and reliability, spiked positive control values were compared with historical value identified for the same amount of miRNAs. The spiked positive controls showed excellent correlation with historical values (p = 0.98) (Fig. 2c). This was a quality control step to evaluate the ability of the assay to distinguish different value of spiked positive control. Our results indicated that assay readouts were sensitive to the amount of miRNA and increase linearly when the amount of spiked in control increased. Of the 68 miRNAs indicated in the inflammatory panel, 63 miRNAs were detectable in our samples (Fig. 2d).

Lack of suitable endogenous control normalizer has been one of the main limitations of using miRNA and EVs associated miRNA diagnostics. Regarding the suitable endogenous control normalizer, miRNA-15b, miRNA-20a and miRNA-15b expression levels showed the lowest intragroup and intergroup variability, with the stability value of 0.189 and 0.232 respectively, according to NormFinder algorithm (Table 1). In RT-qPCR analysis, miRNA-15b consistently showed minimal variability in expression levels among different group of experiments.

**Table 1:** Stability values of different miRNAs based on NormFinder

Gene name	Stability value	Norm Finder ranking
miRNA-15b	0.189	1
miRNA-20a	0.321	3
miRNA-29a	0.405	4
Best combination of two genes		
miRNA-20a and miRNA-15b	0.232	2

Group identifier	EVs isolated from ethanol-fed mice	EVs isolated from pair-fed mice
Intra-group variation		
miRNA-15b	0.101	0.092
miRNA-20a	0.231	0.412
miRNA-29a	0.321	0.249
Intergroup variation		
miRNA-15b	0.003	0.007
miRNA-20a	0.201	0.310
miRNA-29a	0.241	0.379

### Differential miRNA signature of alcohol-fed mice versus control mice

**Fig. 3:**
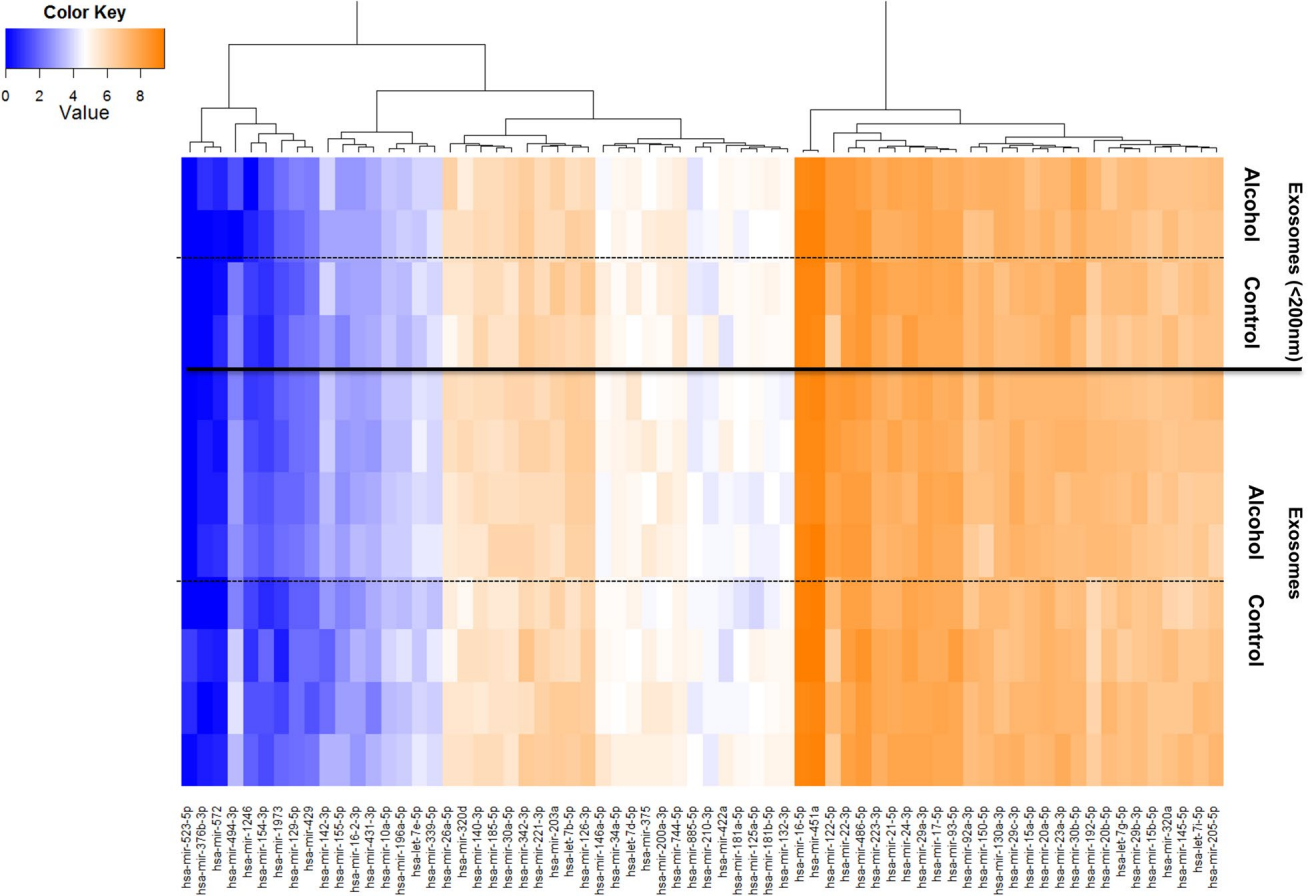
Heat map of differentially expressed EV-associated miRNAs in alcohol-fed mice versus pair-fed mice. Heat map of differentially expressed miRNAs in exosomes isolated from alcohol-fed mice (alcohol exosomes) compared to exosomes isolated from pair-fed mice (control exosomes) using Firefly biowork miRNA assay.

Exosomes were isolated from the sera of 12 chronic alcohol-fed mice and 12 pair-fed mice. To obtain enough exosome-associated total RNA for running microarrays and subsequent qPCR validation, we pooled two samples randomly for a total of six samples in each control and alcohol-fed group. In two samples from the alcohol-fed pair-fed groups, respectively, we isolated the smaller subpopulation of exosomes with the diameter of less than 200 nm, to compare the differential signature between the whole population of exosomes and smaller subpopulation of exosomes. miRNA analysis of microarray data showed no significant difference in miRNA signature between total exosomes and smaller subpopulation of exosomes (with diameter less than 200 nm) in mice sera (Fig. 3). This is in accordance with our previous findings regarding the presence of homogenous exosomes in mice sera [27].

**Fig. 4:**
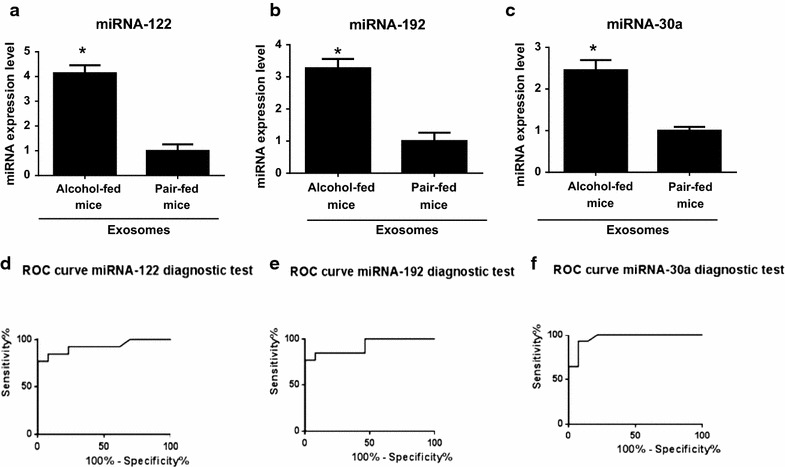
miRNA signatures of serum-derived exosomes in alcohol-fed mice and pair-fed mice and receiver operating characteristic (ROC) curves. Graph demonstrating confirmatory TaqMan miRNA qPCR assay for quantification of **a** miRNA-122, **b** miRNA-192 and **c** miRNA-30 levels in serum-derived exosome isolated from alcohol-fed mice compared to pair-fed mice. Curve of receiver operating characteristic (ROC) analysis constructed using differentially expressed **d** miRNA-122, **e** miRNA-192, and **f** miRNA-30a for discriminating alcohol-fed mice versus control mice.

The heat map and the list of deregulated miRNAs in EVs isolated from alcohol-fed and pair-fed mice are shown in Fig. 3 and Table 2 respectively. Confirmatory qPCR using miRNA-15b as endogenous normalizer and identical amount of exosomal RNA input, showed that miRNA-192, miRNA-122, and miRNA-30a levels in EVs isolated from sera of alcohol-fed mice were significantly increased in alcohol-fed mice compared to the control pair-fed mice (Fig. 4a). In addition, miRNA-744 (1.49-fold), miRNA-1246 (2.40-fold), miRNA-30b (1.50-fold) demonstrated a significant increases in alcohol-fed mice compared to the pair-fed mice (p < 0.05).

**Table 2:** Differential miRNA signature of extracellular-vesicles derived from sera of chronic alcohol-fed mice compared to the pair-fed mice

Probes	Fold changes	P value*
Increased miRNAs in alcohol-fed mice EVs vs paired-fed mice EVs		
miRNA-192	3.29	<0.001
miRNA-122	4.78	0.001
miRNA-30a	1.96	0.001
miRNA-744	1.53	0.009
miRNA-1246	2.90	0.037
miRNA-30b	1.66	0.040
miRNA-130a	1.85	0.042
Decreased miRNAs in alcohol-fed mice EVs vs paired-fed mice EVs		
miRNA-132	1.44	0.012
miRNA-494	1.16	0.011

* P-value obtained from t-test.

### miRNA-192, miRNA-122 and miRNA-30a accurately discriminate the alcohol-fed and control mice

Consistent with the highly significant increase of miRNA-122, miRNA-30a, and miRNA-192 (Fig. 4a–c), those miRNAs showed promising diagnostic values. To test the diagnostic value of the identified miRNAs, we performed Receiver–Operator Characteristic (ROC) curves analysis, and we found that miR-192 has the highest area under the curve (AUC = 0.96; p < 0.001). miRNA-122 and miRNA-30a showed an AUC of 0.92 (p < 0.001) and 0.85 (p < 0.05), respectively (Table 3; Fig. 4d–f).

**Table 3:** ROC curve analysis indicating diagnostic value of each miRNA

	AUC (area under the curve)	Standard error	P value
EVs from alcohol-fed mice vs paired-fed mice			
miRNA-122	0.9260	0.05487	P < 0.001
miRNA-192	0.9668	0.03007	P < 0.001
miRNA-30a	0.8532	0.08031	P < 0.05
EVs from alcoholic hepatitis patients vs control individuals			
miRNA-192	0.95	0.8–1	p < 0.001
miRNA-30a	0.58	0.1589	P = 0.56

### Increased number of EVs in plasma of alcoholic hepatitis patients and characteristic miRNA profile of EVs

**Fig. 5:**
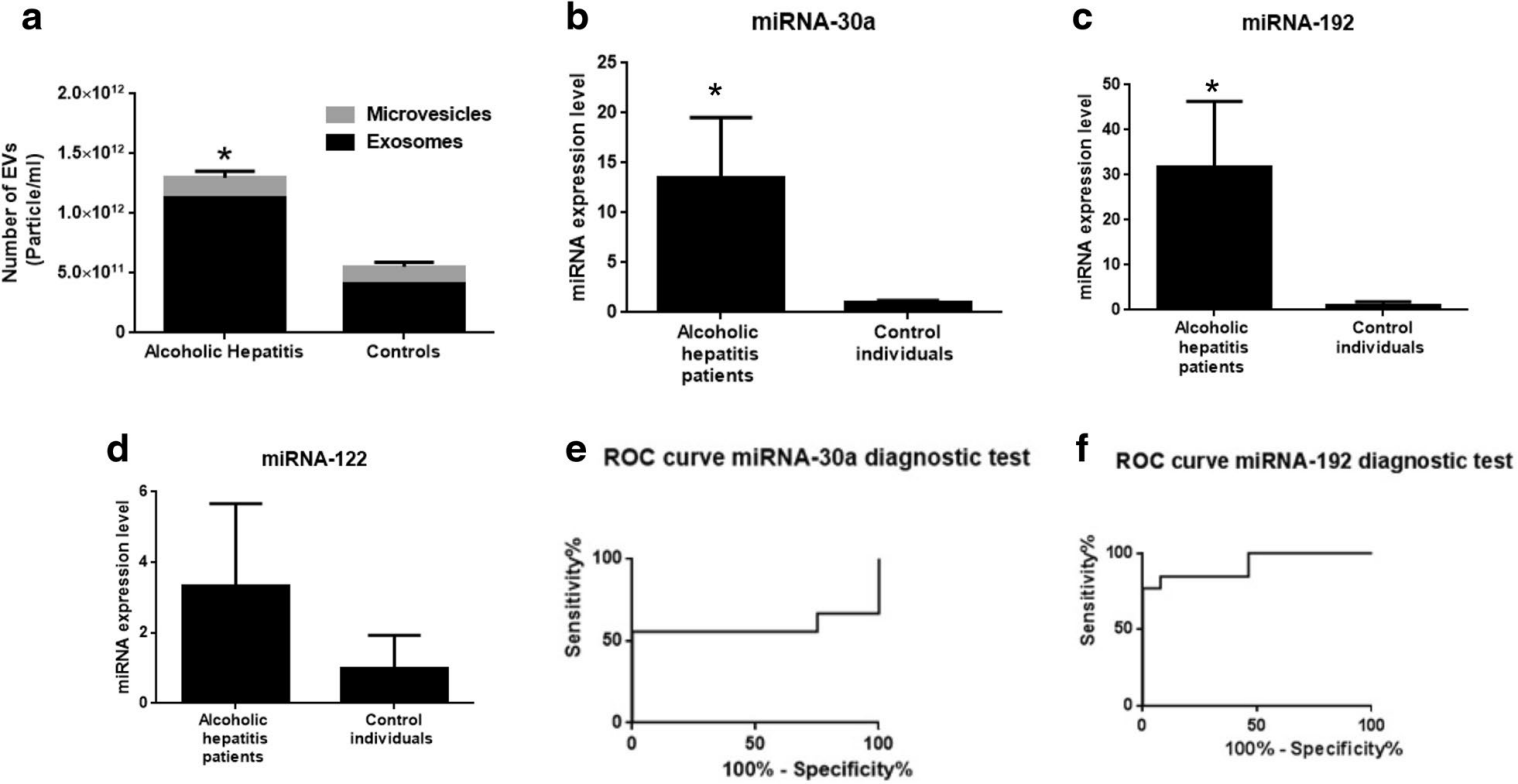
EVs/exosomes characterization and signature in patients with alcoholic hepatitis (AH) versus controls and receiver operating characteristic (ROC) curves. **a** Number of EVs was measured in the plasma of patients with alcoholic hepatitis and healthy individuals using a Nanoparticle tracking analysis (Nanosight). The EVs with diameters less than 150 nm were defined as exosomes and EVs with diameter more than 150 nm were defined as microvesicles. **b**–**d** Levels of miRNA-122, miRNA-192 and miRNA-30 were quantified in plasma-derived EVs isolated from alcoholic hepatitis individuals and healthy controls by TaqMan qPCR assay. **e**, **f** Curve of receiver operating characteristic (ROC) analysis constructed for differentially expressed miRNA-30a, miRNA-192, and for discriminating patients with alcoholic hepatitis versus controls (*p < 0.05).

**Fig. 6:**
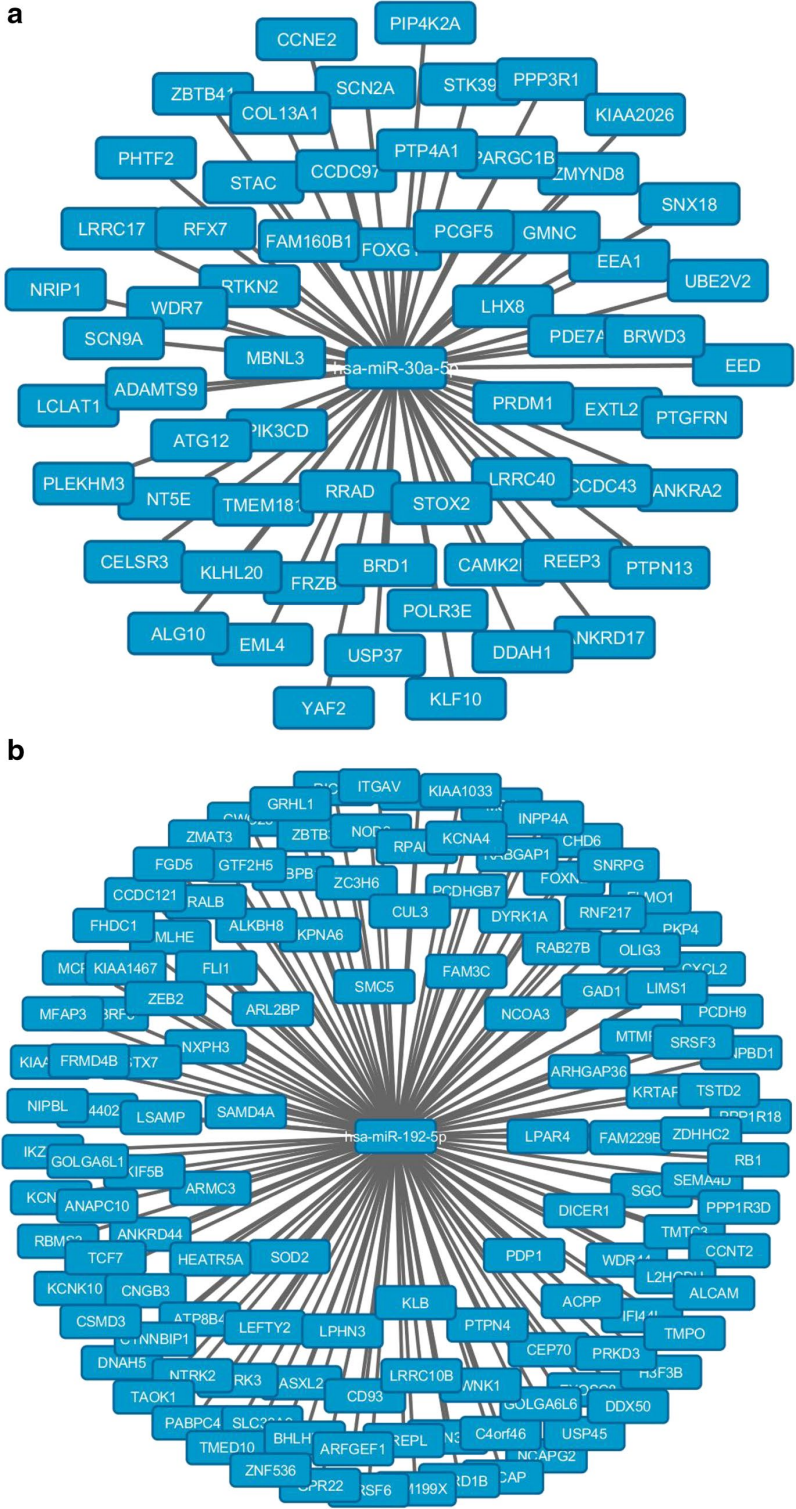
Target prediction for miRNA-30a and miRNA-192. **a** Potential targets of miRNA-30a. **b** Potential targets of miRNA-192.

We evaluated the number and profile of EVs in patients’ plasma (n = 14) and found a significant increase in the number of EVs in the plasma of patients with alcoholic hepatitis, compared to healthy controls (Fig. 5a). The mean age for patients with alcoholic hepatitis and control subjects were 46.28 (SD 6.43) and 28.9 (SD 3.03), respectively. The baseline characteristics of patients are shown in the Additional file 1: Table S1. Consistent with the animal study, most of the circulating EVs in plasma of alcoholic hepatitis patients were in the range of exosomes (less than 150 nm) (Fig. 5a). The level of miRNA-30a and miRNA-192 showed elevated levels in exosomes in patients with alcoholic hepatitis compared to healthy controls (Fig. 5b, c). miRNA-122 showed elevated levels in exosomes isolated from patients with alcoholic hepatitis, but it did not reach statistical significance (Fig. 5d). Based on ROC curve analysis, exosome-associated miRNA-192 can act as an accurate diagnostic test for discrimination of liver disease in the population (AUC = 0.95; p < 0.001) (Fig. 5e, f; Table 3). Using target prediction algorithms, we identified the predicted target protein networks of human miRNA-192 and miRNA-30a (Fig. 6a, b). The first thirty ranked target genes of miRNA-192 and miRNA-30a, as well as gene description are shown in Additional file 1: Tables 2 and 3.

## Discussion

Extracellular vesicles, including microvesicles, exosomes and apoptotic bodies are released from almost all cell types into the microenvironment, and involved in physiological function and the pathology of different diseases [28]. In this study, we found a significantly increased number of circulating EVs in sera of alcohol-fed mice compared to pair-fed mice. Similarly, we observed a significantly increased number of EVs in plasma of AH patients, compared to healthy controls. Interestingly, the vesicles in both animal model and human subjects were mostly in the size range of exosomes; the smaller subpopulation of vesicles. Although there are a few reports that address the roles of EVs in various liver diseases [27, 29–31], this is the first report regarding increased numbers of EVs in alcoholic hepatitis patients.

We found a strong linear correlation between exosome numbers and ALT levels, indicating that the exosomes machinery is affected and an increase in exosome numbers mirrors the hepatocellular damage induced by ethanol. Exosomes originate specifically from secretory multivesicular endosome bodies (MVBs). It has been shown that exosome biogenesis can be ESCRT-complex dependent or sphingomyelinase dependent. Tetraspanins, which are highly enriched in the MVBs, have often been associated with exosomes; smaller sub-fraction of EVs [32, 33]. In this study we observed an increased number of exosomes in the plasma of patients with alcoholic hepatitis and sera of mice after chronic alcohol feeding, which indicate the activation of exosome biogenesis pathways. The fact that we did not observe a considerable amount of microvesicles in models may indicate that the ethanol exposure induces cell stress below the threshold stress for inducing cell death [34]. From the evolutionary biology point of view, increased amount of exosomes shedding after alcohol assault may indicate evolutionary plasticity of the cells for limiting the stressor constrain.

While the mechanism of the EVs biogenesis varies in different subpopulations, genetic materials were detected in all type of vesicles [35]. Analysis of genetic material secreted within different subpopulation of EVs in circulation represents a unique opportunity for diagnosis and therapeutic monitoring of alcoholic hepatitis. Recent evidence suggests that circulating miRNAs can exist in two distinct forms: (1) as free floating EVs where they form a complex with Argonaute 2, the RNA induced silencing complex (RISC), or high density lipoproteins and (2) as packaged within the EVs/exosomes [36, 37]. We found that total RNA is more abundant in the exosome fraction compared to the non-exosome fraction. Exosome-based diagnosis confers several advantages for biomarker discovery compared to conventional strategies involving whole blood analysis. First, exosomes contain RNA, lipids, and proteins that are reflective of the status of parental cells and disease-specific protein or RNA, which are often detected in exosomes [35–37]. Second, the contents inside exosomes are protected by a lipid bilayer of exosomes, which leads to better stability of bio-macromolecules. Third, exosomes are very stable and can be stored for extended period of time. Fourth, exosome-based analysis reduces the complexity of biofluids thereby aiding in the more specific and more sensitive detection of low abundance bio-macromolecules [15, 38, 39]. Although different exosome isolation methods may yield varying results, using a validated established protocol for isolation demonstrates great reproducibility. We used a combination of serial filtration and ExoQuick for isolation of exosomes which has shown reproducibility and efficiency for quantitative studies [40].

We found nine deregulated inflammatory miRNAs in exosomes of alcohol-fed mice. Particularly, miRNA-122, miRNA-30a, and miRNA-192 showed the most substantial increases and revealed an excellent diagnostic value for differentiating alcohol-fed mice versus pair-fed mice. Importantly, of these miRNAs in the cohort of patients with alcoholic hepatitis, we found a significantly elevated level of miRNA-30a and miRNA-192 in the EV-fraction of plasma. This observation in human AH validated our findings in the animal model and indicates that the chronic Lieber DeCarli ethanol feeding model partially presents similar features of human AH. We found elevated levels of miRNA-122 in the circulation in the Lieber–DeCarli dietary mouse model. In contrast, we did not observe statistically significant elevation in miRNA-122 in exosomes isolated from sera of patients with alcoholic hepatitis, which may indicate the difference of the Lieber DeCarli alcohol model with alcoholic hepatitis in patients. This result might be attributed to the patients’ heterogeneity in terms of being in different stages of alcoholic hepatitis or having different kinetics of miRNA-122 in alcohol-induced liver injury.

miRNA-192 showed consistently elevated levels in both alcohol-fed mice samples and in patient- derived alcoholic hepatitis samples. miRNA-192 is the second most abundant miRNA in the human liver and considered as one of the liver specific miRNA constituting around 16.9% of the miRNome in normal liver tissue [41]. In spite of the high abundance in the liver, its regulation in the liver is tightly controlled and was not changed in hepatocellular carcinoma and viral hepatitis [41]. In a study done by our group [42], we showed differential distribution of miRNAs in exosomes and exosome-depleted serum fraction in different types of liver injury. In particular, in alcoholic hepatitis and in inflammatory liver injury, serum/plasma miRNA-122 and miRNA-155 were predominantly associated with the exosome-rich fraction.

We showed that miRNA-192 levels can be used to differentiate plasma from alcoholic hepatitis patients and plasma from controls. Importantly, our results were validated on plasma samples from several individuals. Interactome analysis of miRNA-192 revealed activation of Smad signaling and ZEB proteins, which are related to alcoholic hepatitis pathobiology through regulation of TGFβ/Smad signaling [43]. Another target of miRNA-192 that showed overlap with known pathways of alcoholic hepatitis is Jak2/Arhgef1 signaling pathway, which is correlated with severity of liver disease [44].

The finding of elevated levels of miRNAs species in exosomes after alcohol administration from other tissues such as miRNA-30a, which is highly expressed in heart cells [45], may further demonstrate the effect of alcohol on multiple organs and cell types despite the majority of pathological damage being reported as confined to the liver. Indeed, chronic alcohol administration is associated with direct cardiomyopathy and anemia-induced cardiomyopathy, both in humans and animal models [46, 47]. The diverse origin of exosome-associated miRNAs also suggests that using circulating miRNA as a specific window into the physiopathological condition of the whole effects of alcohol, perhaps in concert with certain other markers profile, will be a powerful approach for diagnosis. However, more data regarding specificity of miRNA-192 and miRNA-30a for alcoholic hepatitis compared to the other types of liver disease, and side by side comparison of those miRNA markers with other markers of liver injury such as ALT should be gathered in future studies. Although we found significant discriminatory ability of exosomal miRNA-192 for alcoholic hepatitis, our study is in the discovery phase and has limited sample size. Further investigations are warranted to confirm our findings in larger cohorts of patients and comparing miR-192 with established scores such as MELD and Maddrey scores.

## Conclusions

In this study we showed that the total number of circulating extracellular vesicles is significantly increased in alcohol-fed mice and human subjects with alcoholic hepatitis. Those EVs were in the range of smaller sub-population of EVs, called exosomes. Comparing the miRNA signature of exosomes from alcohol-fed mice with pair-fed mice showed deregulation of nine inflammatory miRNAs including miRNA-122, miRNA-192 and miRNA-30a. Of those, three miRNA were significantly up-regulated in alcohol-fed mice compared to pair-fed mice and had valuable diagnostic values. Consistently, miRNA-30a and miRNA-192 were increased significantly in exosomes isolated from plasma of alcoholic hepatitis patients.

## Additional file

**Additional file 1:** **Figure S1.** Histological and molecular changes in Lieber-DeCarli mice model. **Table S1.** Characteristics of patients with alcoholic hepatitis. **Table S2.** Predicted targets of miRNA-192. **Table S3.** Predicted targets of miRNA-30a.
