# Supplementary material for: Increased number of circulating exosomes and their microRNA cargos are potential novel biomarkers in alcoholic hepatitis
Source: J Transl Med. 2015 Aug 12;13:261. doi: 10.1186/s12967-015-0623-9 (PMC4533956; doi:10.1186/s12967-015-0623-9)
Supplement: Supplementary file 1 — Additional file 1: Figure S1. Histological and molecular changes in Lieber-DeCarli mice model. Table S1. Characteristics of patients with alcoholic hepatitis. Table S2. Predicted targets of miRNA-192. Table S3. Predicted targets of miRNA-30a. [file 12967_2015_623_MOESM1_ESM.pdf]

Additional File

Increased number of circulating exosomes and their microRNA cargos are potential novel biomarkers in alcoholic hepatitis

Authors: Fatemeh Momen Heravi*, Banishree Saha*, Karen Kodys, Donna Catalano, Abhishek Satishchandran, Gyongyi Szabo
University of Massachusetts Medical School, Worcester, MA

Additional File

Additional File 1: Figure S1. Histological and molecular changes in Lieber-DeCarli mice model. **Table S1.** Characteristics of patients with alcoholic hepatitis. **Table S2.** Predicted targets of miRNA-192. **Table S3.** Predicted targets of miRNA-30a.

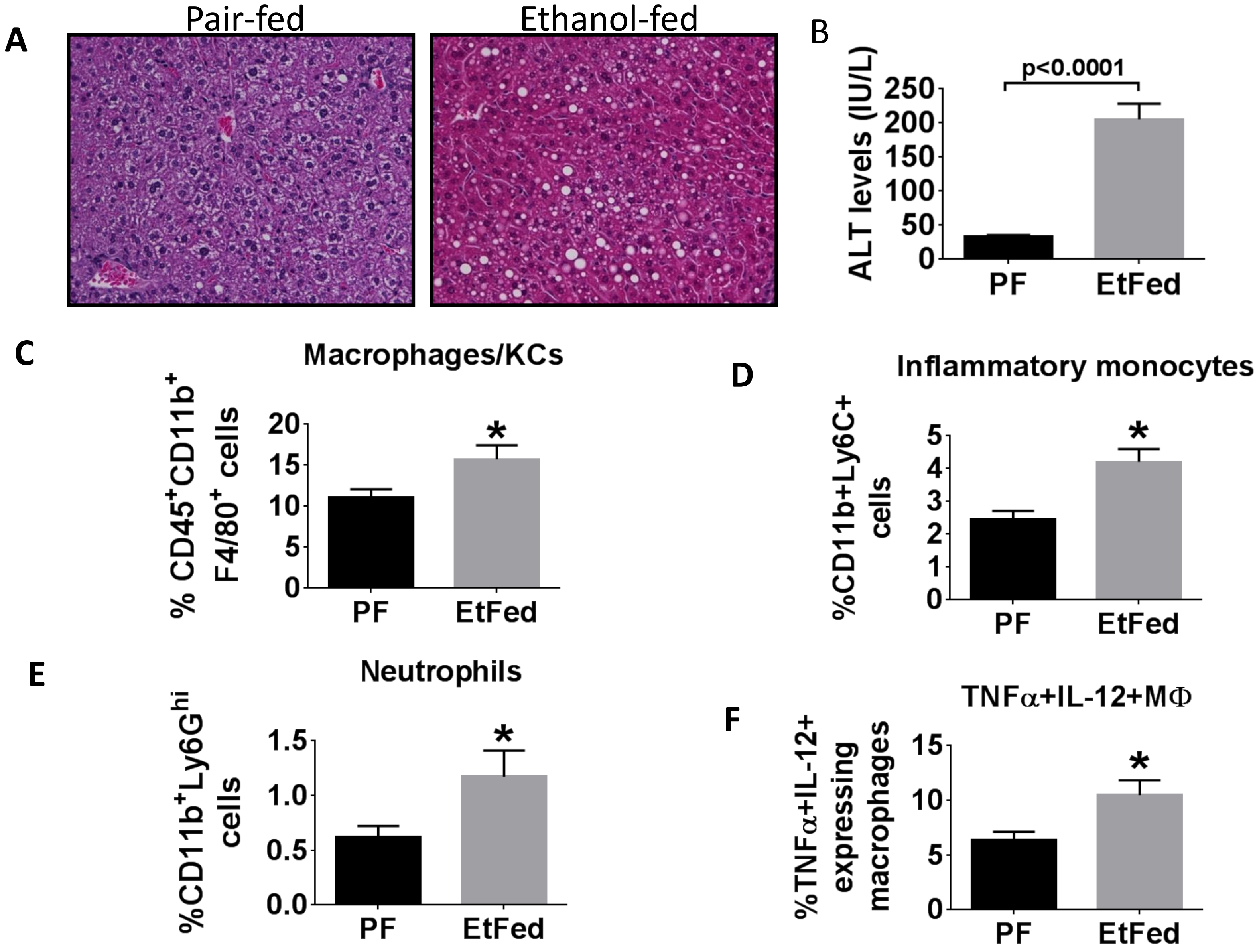

Supplementary Figure 1- Histological and molecular changes in Lieber-DeCarli mice model. Mice were fed control (pair-fed) or alcohol (EtOH-fed) diet for 4 weeks. (A) Liver sections were stained by hematoxylin-eosin stain. (B) ALT was significantly increased in sera of alcohol-fed mice compared to the pair-fed mice. (C) Subpopulation of macrophages/Kupffer cells (KCs), (D) inflammatory monocytes, (E) neutrophils and (F) TNFα and IL-12 positive macrophages increased significantly in alcohol-fed mice compared to the pair-fed mice. The data is represented as Mean ± SE (* indicates $p < 0.05$).

Supplementary Table 1- Characteristics of patients with alcoholic hepatitis

Gender	age (yr)	AST (U/L)	ALT (U/L)	Biliru-bin (mg/dL)	WBC (th/mm3)	RBC (th/mm3)	Hemo-globin (g/dL)	Hemato-crit (%)	Platelets (th/mm)	Segmented neutrophils (%)	Monocytes (%)	Eosinophils (%)	MELD Score
Male	48	153	88	13.3	11.2	2.94	10.7	30.8	219	72	11	2	18
Female	54	82	44	0.7	7.4	4.13	14.4	40.7	241	62.7	5.6	0	10
Male	38	104	139	6.9	7.6	3.69	12.3	36.8	191	78	11	1	15
Male	46	42	20	4.2	2.4	2.47	8.4	24.5	32	53.3	8.9	4.1	16
Female	60	28	23	2.4	6.1	3.9	13	38.7	178	60	11	n/a	11
Male	53	27	14	0.4	9.7	4.34	13.8	40.7	311	71.3	6.2	2.4	7
Female	38	131	52	2.1	5.2	4.24	14.7	43.8	124	54.5	4.7	3.9	10
Male	48	79	25	14.4	7.7	2.42	9.1	26.8	82	85	4	n/a	25
Male	49	33	13	1.8	3.1	3.64	9.1	27.9	87	65.2	21.5	1	10
Male	48	34	12	1.4	11.1	2.35	7.8	23.4	140	79.4	6.6	0.2	12
Female	39	166	28	1.8	5.8	3.46	12.3	35.9	64	62	10	6.1	12
Male	38	108	58	1.7	4.6	4.44	14.9	43.4	66	49.1	10.8	2.2	10
Male	46	133	51	21.3	16.6	4.26	12.6	38.4	90.2	75	15.9	0.5	23
Female	43	157	43	16.1	14	2.01	8	22.6	127	87.3	4.8	1	22

Supplementary Table 2- Predicted targets of miRNA-192

Target Rank	Target Score	Gene Symbol	Gene Description
**1**	98	EREG	epiregulin
**2**	96	DYRK3	dual-specificity tyrosine-(Y)-phosphorylation regulated kinase 3
**3**	94	LPAR4	lysophosphatidic acid receptor 4
**4**	94	ZEB2	zinc finger E-box binding homeobox 2
**5**	94	MSN	moesin
**6**	94	ARFGEF1	ADP-ribosylation factor guanine nucleotide-exchange factor 1 (brefeldin A-inhibited)
**7**	92	CCNT2	cyclin T2
**8**	91	PDP1	pyruvate dehyrogenase phosphatase catalytic subunit 1
**9**	91	GPR22	G protein-coupled receptor 22
**10**	91	DICER1	dicer 1, ribonuclease type III
**11**	89	ANAPC10	anaphase promoting complex subunit 10
**12**	89	LIMS1	LIM and senescent cell antigen-like domains 1
**13**	87	WDR44	WD repeat domain 44
**14**	86	RPAP2	RNA polymerase II associated protein 2
**15**	85	FRMD4B	FERM domain containing 4B
**16**	84	ARL2BP	ADP-ribosylation factor-like 2 binding protein
**17**	84	STX7	syntaxin 7
**18**	84	CNGB3	cyclic nucleotide gated channel beta 3
**19**	84	BHLHE22	basic helix-loop-helix family, member e22
**20**	83	FGD5	FYVE, RhoGEF and PH domain containing 5
**21**	81	RB1	retinoblastoma 1
**22**	80	KIAA1033	KIAA1033
**23**	79	RNF217	ring finger protein 217
**24**	79	TMTC3	transmembrane and tetratricopeptide repeat containing 3
**25**	78	CXCL2	chemokine (C-X-C motif) ligand 2
**26**	78	NCOA3	nuclear receptor coactivator 3
**27**	78	TMPO	thymopoietin
**28**	78	PRKD3	protein kinase D3
**29**	78	KIAA1467	KIAA1467
**30**	77	C4orf46	chromosome 4 open reading frame 46

Supplementary Table 3- Predicted targets of miRNA-30a

Target Rank	Target Score	Gene Symbol	Gene Description
**1**	100	TNRC6A	trinucleotide repeat containing 6A
**2**	100	YAF2	YY1 associated factor 2
**3**	100	POLR3E	polymerase (RNA) III (DNA directed) polypeptide E (80kD)
**4**	100	PHTF2	putative homeodomain transcription factor 2
**5**	100	ANKRA2	ankyrin repeat, family A (RFXANK-like), 2
**6**	100	LHX8	LIM homeobox 8
**7**	100	CELSR3	cadherin, EGF LAG seven-pass G-type receptor 3
**8**	100	EED	embryonic ectoderm development
**9**	100	WDR7	WD repeat domain 7
**10**	100	BRWD3	bromodomain and WD repeat domain containing 3
**11**	100	SCN2A	sodium channel, voltage-gated, type II, alpha subunit
**12**	100	KLHL20	kelch-like family member 20
**13**	100	EEA1	early endosome antigen 1
**14**	100	RFX7	regulatory factor X, 7
**15**	100	PLEKHM3	pleckstrin homology domain containing, family M, member 3
**16**	100	NT5E	5'-nucleotidase, ecto (CD73)
**17**	99	ANKRD17	ankyrin repeat domain 17
**18**	99	STAC	SH3 and cysteine rich domain
**19**	99	STK39	serine threonine kinase 39
**20**	99	PDE7A	phosphodiesterase 7A
**21**	99	PCGF5	polycomb group ring finger 5
**22**	99	GMNC	geminin coiled-coil domain containing
**23**	99	EML4	echinoderm microtubule associated protein like 4
**24**	99	RRAD	Ras-related associated with diabetes
**25**	99	PPARGC1B	peroxisome proliferator-activated receptor gamma, coactivator 1 beta
**26**	99	MBNL3	muscleblind-like splicing regulator 3
**27**	99	PIP4K2A	phosphatidylinositol-5-phosphate 4-kinase, type II, alpha
**28**	99	SCN9A	sodium channel, voltage-gated, type IX, alpha subunit
**29**	99	PTP4A1	protein tyrosine phosphatase type IVA, member 1
**30**	99	LRRC40	leucine rich repeat containing 40
